# *Anemarrhena asphodeloides* Bunge and its constituent timosaponin‐AIII induce cell cycle arrest and apoptosis in pancreatic cancer cells

**DOI:** 10.1002/2211-5463.12457

**Published:** 2018-06-11

**Authors:** Catherine B. MarElia, Arielle E. Sharp, Tiffany A. Shemwell, Y. Clare Zhang, Brant R. Burkhardt

**Affiliations:** ^1^ Department of Cell Biology, Microbiology and Molecular Biology University of South Florida Tampa FL USA; ^2^ Practice of Oriental Medicine Tucson AZ USA

**Keywords:** *Anemarrhena asphodeloides*, apoptosis, pancreatic cancer, timosaponin‐AIII

## Abstract

Pancreatic cancer is one of the most recalcitrant and lethal of all cancers. We examined the effects of *Anemarrhena asphodeloides* (AA) and timosaponin‐AIII (TAIII), a steroidal saponin present in AA, on pancreatic cancer cell proliferation and aimed to elucidate their potential apoptotic mechanisms of action. Viability assays and cell cycle analysis revealed that both AA and TAIII significantly inhibited pancreatic cancer cell proliferation and cell cycle progression compared to treatment with gemcitabine, the standard chemotherapeutic agent for advanced pancreatic cancer. We identified a dose‐dependent increase in caspase‐dependent apoptosis and activation of pro‐apoptotic PI3K/Akt pathway proteins, with a subsequent downregulation of pro‐survival PI3K/Akt pathway proteins, in pancreatic cancer cells treated with AA or TAIII over those treated with gemcitabine.

**Abbreviations**
AA: *Anemarrhena asphodeloides*
BAD: Bcl2‐associated agonist of cell death
GSK: glycogen synthase kinase
KRAS: Kirsten rat sarcoma viral oncogene homologue
mTOR: mammalian target of rapamycin
PDAC: pancreatic ductal adenocarcinoma
PI3K: phosphatidylinositol‐3 kinase
PTEN: phosphatase and tensin homologue deleted on chromosome ten
TAIII: timosaponin‐AIII

Pancreatic ductal adenocarcinoma (PDAC) is one of the most lethal cancers with nearly equal incidence and mortality rates due to a combination of factors including a lack of early detection methods, limited surgical options, and ineffective treatment options due to drug and apoptotic resistance 1. Surgery remains the only potential curative therapeutic approach but is not a viable option for most patients as more than 80% are already in an advanced disease state at the time of detection 2. Relapse of the disease is common in patients whose tumors are initially resectable, rendering chemotherapy a necessity even after surgical intervention. Standard chemotherapy for advanced PDAC since 1997 has been the deoxycytidine analog gemcitabine alone or in combination with nab‐Paclitaxel or 5‐fluorouracil 3. However, the prognosis in these patients remains poor due to chemoresistance resulting in substantial treatment failure in addition to debilitating side effects 4.

Mutations within a concerted network of several core cellular signaling pathways can be found throughout the progression of PDAC 5. Phosphatidylinositol‐3 kinase (PI3K)/Akt signaling represents one of the most frequently altered pathways in PDAC and regulates the activity of a litany of substrates involved in cell survival, proliferation, growth, and motility. Increased activity of the serine/threonine kinase Akt promotes cell survival and evasion of apoptosis through the inhibition of pro‐apoptotic proteins including Bcl‐2‐associated death promoter (BAD) and the activation of pro‐survival substrates and has been observed in several types of cancers 6, 7. One of the primary negative regulators of PI3K/Akt activity is phosphatase and tensin homologue deleted on chromosome ten (PTEN); inactivating mutations within PTEN contribute to the hyperactive state of the PI3K/Akt pathway and progression of PDAC 8. Glycogen synthase kinase‐3 (GSK‐3α/β) plays a critical role in controlling several cell cycle regulators and transcription factors which affect cell fate. Akt catalyzes the inhibition of GSK‐3 via phosphorylation at Ser21 and Ser9 (on GSK‐3α and GSK‐3β, respectively) 9. The mammalian target of rapamycin (mTOR) signaling pathway is also affected by mutational Akt activity through phosphorylation and activation of the mTORC1 complex. Phosphorylated mTOR promotes cell growth by enhancing the transcription of growth factor mRNA via the activation of several substrates, including p70 S6 kinase 1 10.

The exploration of plants as a source of chemotherapeutic agents in Western medicine has been active since the 1950s with the discovery of the vinca alkaloids vinblastine and vincristine isolated from *Catharanthus roseus* and the isolation of cytotoxic podophyllotoxins 11. The rhizome of *Anemarrhena asphodeloides* Bunge (AA) has been a significant fixture in Traditional Chinese Medicine for thousands of years and is the only species in the genus. Its primary chemical components are steroidal saponins, flavonoids, phenylpropanoids, alkaloids, steroids, organic acids, and anthraquinones. Most abundant among the identified constituents are steroidal saponins. Timosaponin‐AIII (TAIII) , a steroidal saponin first isolated from AA by Kawasaki *et al*. in 1963, has exhibited pro‐apoptotic and antimetastatic efficacy against non‐small‐cell lung cancer, melanoma, colorectal carcinoma, and breast cancer *in vitro* 12, 13, 14, 15, 16. Although recent studies suggest AA and its constituents may possess potent antitumor properties, its prospective use in the treatment of refractory pancreatic cancer has yet to be elucidated. In this study, we identified the inhibitory effects of AA and TAIII on PDAC cell lines in part through modulation of PI3K/Akt pathway proteins.

## Materials and methods

### Chemicals and reagents

Timosaponin‐AIII (CAS registry number: 41059‐79‐4) was purchased from Santa Cruz Biotechnology (Santa Cruz, CA, USA). HPLC analysis by the manufacturer determined the purity was > 98%. Gemcitabine hydrochloride (CAS registry number: 122111‐03‐9) was purchased from Sigma‐Aldrich (St. Louis, MO, USA). HPLC analysis by the manufacturer determined a purity > 98%. Rabbit monoclonal antibodies against cleaved caspase‐3 (Asp175; Cat. 9664), mouse monoclonal antibody against β‐actin (Cat. 3700), and horseradish peroxidase (HRP)‐linked secondary antibodies were purchased from Cell Signaling Technology (Beverly, MA, USA).

### Preparation of AA extract and TAIII stock

Dried extract granules of AA Bunge rhizome were originally obtained from Tianjiang Pharmaceutical Co. (Jiangsu, China. Batch number 1005417; corresponds to Chinese Pharmacopeia specification) and kindly provided to us by C. Zhang. The dried extract was reconstituted in sterile mQ water at a ratio of 1 : 4 w/v and mixed on a rocker at room temperature for 72 h. The solution was centrifuged at 2000 ***g*** for 10 min to separate undissolved particles and sterilized using a 0.2 μm PEM filter. Total protein content within the extract stock was determined using the Pierce BCA protein assay (Thermo Fisher Scientific Inc., Waltham, MA, USA). Extract stock was stored at 4 °C and diluted with sterile mQ water to the indicated concentration prior to each experiment.

A stock solution of 8 mm TAIII was prepared in DMSO then diluted with sterile mQ water to a final concentration of 0.5% DMSO for each treatment condition. Stock solution was stored at −20 °C.

### Determination of TAIII content in AA extract via LC–MS–TOF

LC–MS analysis was performed using Agilent 1200 series/6230 TOF liquid chromatography/mass spectrometer with a Synergi™ 4 μm Hydro‐RP LC column (250 × 4.6 mm) with 80 Å pore size. Samples of AA (0.5 mg·mL^−1^) and TAIII (0.1 mg·mL^−1^) were run in positive mode at a flow rate of 1 mL per min using a 14‐min gradient of 0–98% acetonitrile in 0.05% formic acid. TAIII content in the AA extract was determined by comparison with reference sample.

### Cell culture

PANC‐1 and BxPC‐3 cells were cultured in growth medium (Dulbecco's modified Eagle's medium with L‐glutamine and RPMI 1640 with l‐glutamine, respectively) supplemented with 10% FBS and 1% penicillin–streptomycin (100 units·mL^−1^ penicillin and 100 μg·mL^−1^ streptomycin). Both PANC‐1 and BxPC‐3 cell lines were authenticated via STR profiling (Promega, Madison, WI, USA) and confirmed to be an exact match to the indicated cell line by ATCC (STR12699 and STR12675). Cells were maintained in a humidified incubator in 5% CO_2_ at 37 °C.

### Cell viability assay

Cell viability was assessed via modified 3‐(4,5‐dimethylthiazol‐2‐yl)‐2,5‐diphenyltetrazolium bromide assay using the CellTiter 96 Non‐Radioactive cell proliferation assay (Promega). Briefly, cells were seeded at 10 000 cells per well in a 96‐well plate and allowed to attach overnight. The cells were then treated with equal volumes of various concentrations of AA and TAIII, with and without 1 mm gemcitabine, 1 mm gemcitabine alone, and sterile mQ water or 0.5% DMSO vehicle control for 24 or 48 h. Absorbance was measured as optical density (OD) at a wavelength of 570 nm using a VersaMax microplate reader (Molecular Devices, LLC. Sunnyvale, CA, USA). The OD of vehicle‐treated control cells represented 100% viability. Viability of treated cells was expressed as a percentage of vehicle‐treated control cells.

### Flow cytometric analysis of cell cycle distribution

Cell cycle distribution was determined using propidium iodide (PI) cellular DNA staining. BxPC‐3 cells were seeded at a density of 1.25 × 10^6^ cells in 5 mL in 25‐cm^2^ flasks and allowed to attach overnight. The media was then replaced with fresh media containing each treatment condition. After 24 h, the cells were harvested and washed then re‐suspended in cold PBS. The cells were added dropwise to cold 70% ethanol and fixed overnight at −20 °C. Fixed cells were washed in cold PBS and filtered through a 40‐μm nylon cell strainer to remove aggregates. The cells were stained at a density of 1 × 10^6^ cells in 500 μL staining solution (0.1% Triton X‐100, 20 μg·mL^−1^ PI, and 0.2 mg·mL^−1^ DNase‐free RNase A in PBS) and incubated at RT in the dark for 30 min. Intracellular DNA data were acquired by a BD Accuri C6 cytometer (Becton Dickinson, San Jose, CA, USA). Debris and doublets were excluded by gating on forward vs. side scatter‐area and forward scatter‐area vs. forward scatter‐height. Gates were performed on the control sample and uniformly applied to each sample. At least 10 000 gated events were used for analysis and the resulting cell cycle distribution was determined using fcs express 6 software (*De Novo* Software, Glendale, CA, USA).

### Protein extraction and Western blot analysis

PANC‐1 cells were seeded at a density of 1.25 × 10^6^ cells in 5 mL in 25‐cm^2^ flasks and treated as indicated above. After collection, standard lysis buffer supplemented with 1× Halt Protease Inhibitor Cocktail (Thermo Fisher Scientific Inc.) was used to obtain whole cell lysate. The samples were sonicated, and protein concentrations were determined using the BCA Protein Assay. Equivalent protein content was loaded into each lane, separated by SDS/PAGE (Mini‐PROTEAN TGX Pre‐Cast gels, 4–20%), and transferred to PVDF membranes. Membranes were blocked with 5% nonfat dry milk in Tris‐buffered saline with 0.05% Tween‐20 (TBST) and probed with primary antibodies against indicated proteins diluted in 5% nonfat dry milk or BSA in TBST overnight at 4 °C. Blots were then incubated with a HRP‐conjugated secondary antibody specific to the primary antibody in 5% nonfat dry milk in TBST at RT for 1 h. Signal was detected using SuperSignal West Pico PLUS Chemiluminescent Substrate (Pierce, Rockford, IL, USA). Blots were imaged and analyzed using the Amersham Imager 600 and the accompanying imagequant tl 8.1 software (GE Healthcare Life Sciences, Pittsburgh, PA, USA).

### Analysis of PI3K/Akt pathway signaling activity via bead‐based multiplex assay

Phosphorylated forms of Akt (Ser^473^), mTOR (Ser^2248^), BAD (Ser^136^), p70 S6 kinase (Thr^389^), GSK‐3α/β (Ser^21^/Ser^9^), and PTEN (Ser^380^) were detected in the lysate of PANC‐1 cells treated under the aforementioned conditions using the Bio‐Plex Pro cell signaling Akt panel (Bio‐Rad, Hercules, CA, USA). The only deviation from the given protocol was the use of 1× Halt Protease Inhibitor Cocktail in place of phenylmethylsulfonyl fluoride in the cell lysis buffer. Analysis was performed using the Luminex MAGPIX (Luminex, Austin, TX, USA). Percent median fluorescence intensity (MFI) of each analyte is expressed as a percentage of the MFI corresponding to the vehicle control‐treated sample.

### Statistical analysis

Results presented as mean ± SE of the number of indicated independent experiments. Statistical significance was determined by performing a two‐tailed, unpaired Student's *t*‐test or one‐way ANOVA followed by Tukey's multiple comparison post‐test in graphpad prism 5.01 (GraphPad Software, San Diego, CA, USA).

## Results

### TAIII content in AA extract via LC–MS–TOF analysis

**Figure 1:**
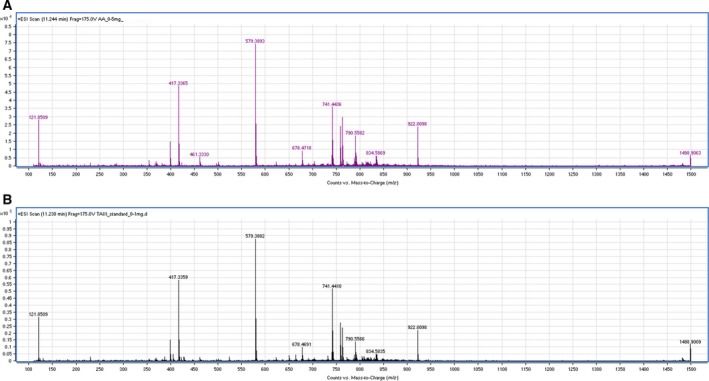
TAIII present in AA extract. The presence of timosaponin‐AIII in our AA extract was determined by comparing the LC–MS–TOF ESI spectra of the whole extract (A) to that of the TAIII reference sample (B).

As TAIII is one of the chemical signatures of AA, and to relatively compare the activity of the whole extract with TAIII as a single compound, we first wanted to confirm the presence of TAIII in our AA extract. We employed LC–MS–TOF to compare the ESI‐MS spectra of the extract (Fig. 1A; RT: 11.24) to that of the TAIII reference standard (Fig. 1B; RT: 11.23). Analysis of the standard TAIII sample and the AA extract showed the protonated molecule at *m/z* 741.44. Furthermore, fragment ions at *m/*z 579.38 [MH – Glu]^+^ and 417.34 [MH – Glu – Gal]^+^ characteristic of TAIII are present in both ESI‐MS spectra 17.

### AA enhances inhibition of pancreatic cancer cells by gemcitabine

**Figure 2:**
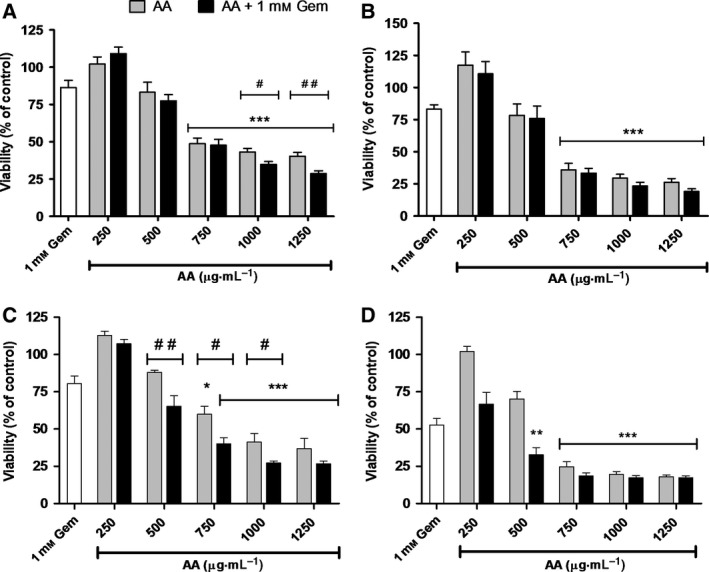
AA inhibits viability of PDAC cell lines and potentiates gemcitabine efficacy. PANC‐1 (A,B) and BxPC‐3 (C,D) cells were incubated with the indicated concentrations of AA, with and without 1 mm gemcitabine, 1 mm gemcitabine alone, or vehicle for 24 (A,C) or 48 (B,D) h. Data from three independent experiments are expressed as mean ± SE. Viability was calculated as a percentage of the vehicle‐treated control cells. Statistically significant inhibition was determined compared to cells treated with gemcitabine alone (*P < 0.05, **P < 0.01, ***P < 0.001) and between treatment with AA alone and AA + gemcitabine (#*P* < 0.05, ##*P* < 0.01).

PANC‐1 and BxPC‐3 cells are resistant to gemcitabine to varying degrees, with PANC‐1 cells exhibiting less sensitivity than BxPC‐3 cells 18. To determine the effect of AA alone and in the presence of gemcitabine on the viability of pancreatic cancer cells, we treated PANC‐1 and BxPC‐3 cells for 24 and 48 h with increasing concentrations of AA or AA and 1 mm gemcitabine. As shown in Fig. 2, AA exhibited a dose‐dependent decrease in viability of both cell lines. The viability of PANC‐1 cells exposed to 750, 1000, and 1250 μg·mL^−1^ AA alone and with gemcitabine for 24 and 48 h was substantially decreased compared to those treated with only gemcitabine (*P* < 0.001)(Fig. 2A,B). Co‐treatment of 1000 and 1250 μg·mL^−1^ AA plus gemcitabine for 24 h was significantly more effective at inhibiting viability than the same concentration of AA alone (*P* < 0.05 or *P* < 0.01; Fig. 2A). A marked decrease in cell proliferation was seen in BxPC‐3 cells treated with AA alone and with gemcitabine at concentrations > 500 μg·mL^−1^ (*P* < 0.05 or *P* < 0.001; Fig. 2C,D). BxPC‐3 cells treated for 24 h with 500, 750, and 1000 μg·mL^−1^ AA plus gemcitabine exhibited significantly less viability than those treated with the same concentration of AA (*P* < 0.05 or *P* < 0.01). Taken together, these data suggest that AA not only inhibits the viability of PANC‐1 and BxPC‐3 cells, but it also appreciably enhances the effect of gemcitabine in a dose‐dependent manner.

### Effects of TAIII on viability of pancreatic cancer cell lines

**Figure 3:**
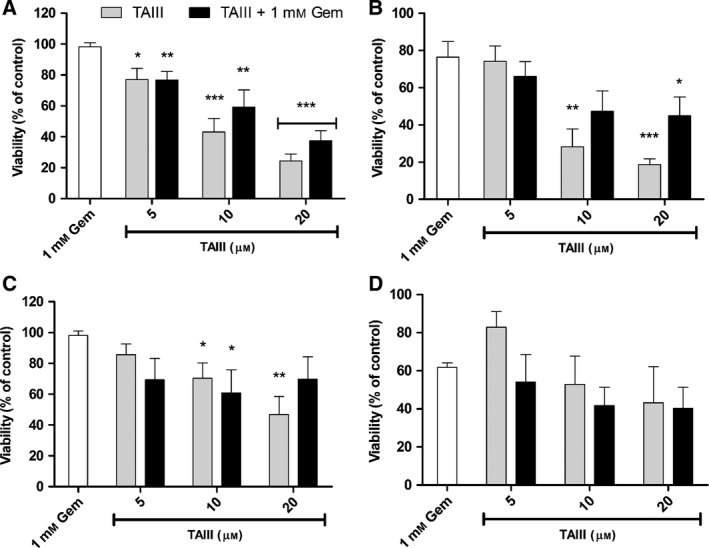
Effect of TAIII on PANC‐1 and BxPC‐3 cell viability. PANC‐1 (A,B) and BxPC‐3 (C,D) cells were incubated with the indicated concentrations of TAIII, with and without 1 mm gemcitabine, 1 mm gemcitabine alone, or vehicle for 24 (A,C) or 48 (B,D) h. Data from three independent experiments are expressed as mean ± SE. Viability was calculated as a percentage of the vehicle‐treated control cells. Statistically significant inhibition was determined compared to cells treated with gemcitabine alone (*P < 0.05, **P < 0.01, ***P < 0.001).

We next wanted to examine the effect of the single compound TAIII on the viability of PANC‐1 and BxPC‐3 cells. As shown in Fig. 3, TAIII dose‐dependently inhibited PANC‐1 cells (Fig. 3A,B), with the highest dose reducing the viability after 24 and 48 h to ~ 25% and 19%, respectively. Viability of PANC‐1 cells treated for 24 h with 5, 10, and 20 μm TAIII was significantly less than in those treated with gemcitabine alone (*P* < 0.05 or *P* < 0.001). Simultaneous exposure of TAIII with gemcitabine also resulted in significant inhibition of PANC‐1 cells compared to gemcitabine, although not to the same degree as the TAIII‐only samples. BxPC‐3 cells treated for 24 h with 10 and 20 μm TAIII alone and 10 μm TAIII with gemcitabine also exhibited significantly reduced viability compared to gemcitabine treatment (*P* < 0.05 or *P* < 0.01; Fig. 3C). There was no significant difference observed in BxPC‐3 cells treated for 48 h with TAIII alone or with gemcitabine compared to gemcitabine alone (Fig. 3D).

### BxPC‐3 cell cycle progression is inhibited by AA and arrested in G1 phase by TAIII

**Figure 4:**
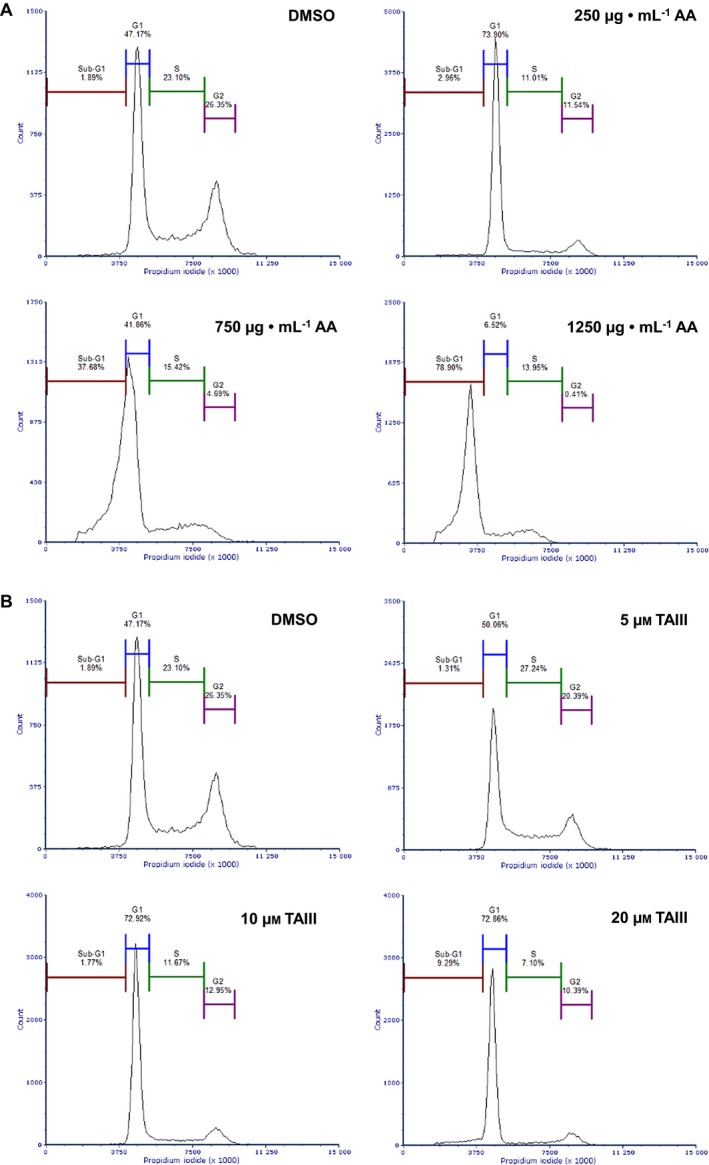
AA and TAIII induce cell cycle arrest and dysfunction in BxPC‐3 cells. Flow cytometric analysis of BxPC‐3 cells treated with the indicated concentrations of AA (A), TAIII (B), or 0.5% DMSO for 24 h. Cell cycle distribution was determined by staining intracellular DNA with PI. At least 10 000 gated cells used for analysis of each sample. Data shown are representative of three independent experiments.

To elucidate the effect of AA and TAIII treatment on the progression of the cell cycle, BxPC‐3 cells treated with increasing concentrations of either AA or TAIII for 24 h were fixed and stained with PI and their DNA content was analyzed by flow cytometry. As shown in Fig. 4A, AA markedly disrupts the cell cycle. At 250 μg·mL^−1^ AA, there is a greater population of cells in G_1_ phase compared to the control sample (73.90% and 47.17%, respectively), allowing fewer cells to reach S and G_2_/M phases. At 750 and 1250 μg·mL^−1^ AA, the cells exhibit a drastic shift in the cycle resulting in a large portion of the cells stalling in the sub‐G_1_ phase (37.68% and 78.90%, respectively) compared to the control (1.89%). Exposure to increasing concentrations of TAIII for 24 h resulted in a marked increase in the population of cells in G_1_ phase (50.06%, 72.92%, and 72.86%) and a subsequent decrease in the S and G_2_/M phase populations compared to the control (Fig. 4B). Cells treated with 20 μm TAIII exhibit a notable increase in the sub‐G_1_ population compared to the control group (9.29% and 1.89%, respectively).

### AA and TAIII improve disruption of BxPC‐3 cell cycle by gemcitabine

**Figure 5:**
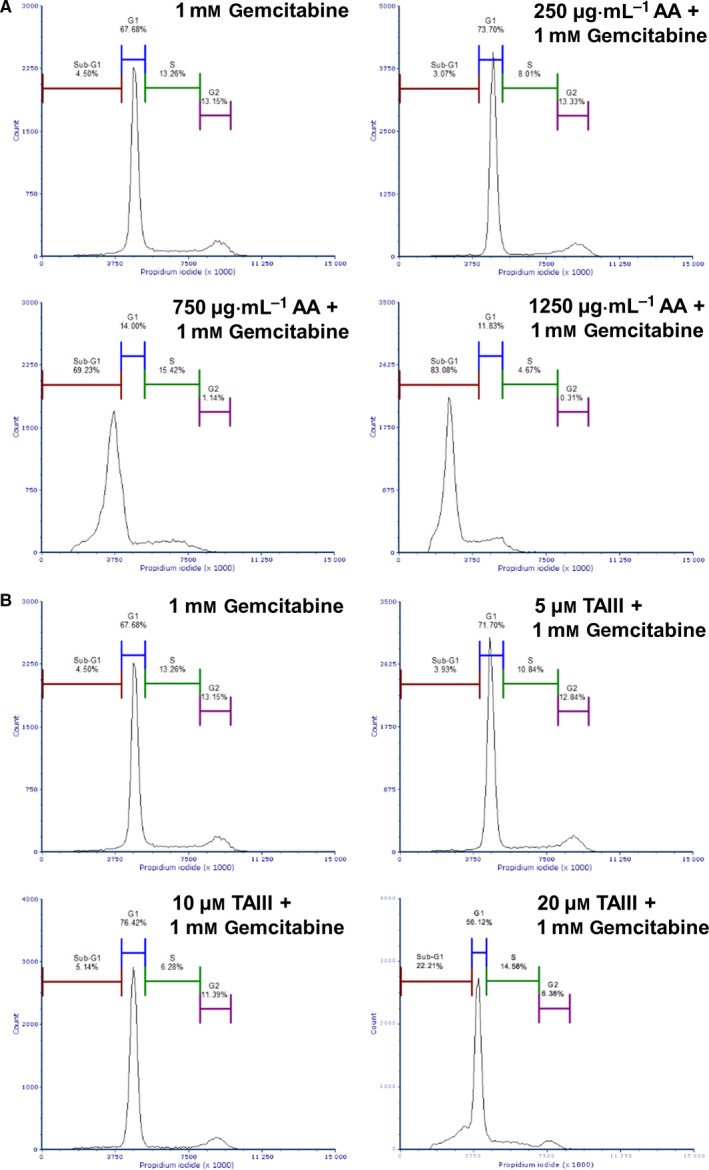
Co‐treatment with AA or TAIII enhances ability of gemcitabine to impede BxPC‐3 cell cycle progression. Flow cytometric analysis of BxPC‐3 cells treated with the indicated concentrations of AA + 1 mm gemcitabine (A), TAIII + 1 mm gemcitabine (B), or 1 mm gemcitabine alone for 24 h. Cell cycle distribution was determined by staining intracellular DNA with PI. At least 10 000 gated cells used for analysis of each sample. Data shown are representative of three independent experiments.

Pancreatic cancer is frequently resistant to gemcitabine, which can be due to several possible mechanisms including efflux from the cell, downregulation of gemcitabine‐activating proteins, and upregulation of gemcitabine‐inactivating proteins 19. We therefore wanted to determine whether either AA or TAIII co‐treatment with gemcitabine could have a greater effect on cell cycle progression than gemcitabine alone. Exposure to AA and gemcitabine dramatically increased the portion of cells in the sub‐G_1_ phase when compared to gemcitabine alone (Fig. 5A). The co‐treated cells also resulted in a more dramatic shift from the G_1_ to sub‐G_1_ populations than those treated with the corresponding concentration of AA alone (Fig. 4A). TAIII plus gemcitabine resulted in a dose‐dependent arrest in G_1_ phase and subsequent increase in the sub‐G_1_ population compared to the gemcitabine‐only treated cells (Fig. 5B). As with AA, co‐treatment with TAIII and gemcitabine resulted in a greater population of cells in the sub‐G_1_ phase than those treated with TAIII alone (Fig. 4B). Table 1 summarizes the data from Figs 4 and 5. These results indicate that co‐treatment of AA or TAIII and gemcitabine is more effective at inducing BxPC‐3 cell cycle arrest than treatment with AA, TAIII, or gemcitabine as a single agent.

**Table 1:** Percentage of total gated cells in each population

Treatment	Sub‐G_1_	G_1_	S	G_2_/M
DMSO	1.89	47.17	23.10	26.35
AA 250 μg·mL^−1^	2.96	73.90	11.01	11.54
AA 750 μg·mL^−1^	37.68	41.86	15.42	4.69
AA 1250 μg·mL^−1^	78.90	6.52	13.95	0.41
TAIII 5 μm	1.31	50.06	27.24	20.39
TAIII 10 μm	1.77	72.92	11.67	12.95
TAIII 20 μm	9.29	72.86	7.10	10.39
Gemcitabine 1 mm	4.50	67.68	13.26	13.15
AA 250 μg·mL^−1^ + 1 mm gem	3.07	73.70	8.01	13.33
AA 750 μg·mL^−1^ + 1 mm gem	69.23	14.00	15.42	1.14
AA 1250 μg·mL^−1^ + 1 mm gem	83.08	11.83	4.67	0.31
TAIII 5 μm + 1 mm gem	3.93	71.70	10.84	12.84
TAIII 5 μm + 1 mm gem	5.14	76.42	6.28	11.39
TAIII 5 μm + 1 mm gem	22.21	56.12	14.58	6.38

### AA and TAIII modulate activation of PI3K/Akt pathway members

**Figure 6:**
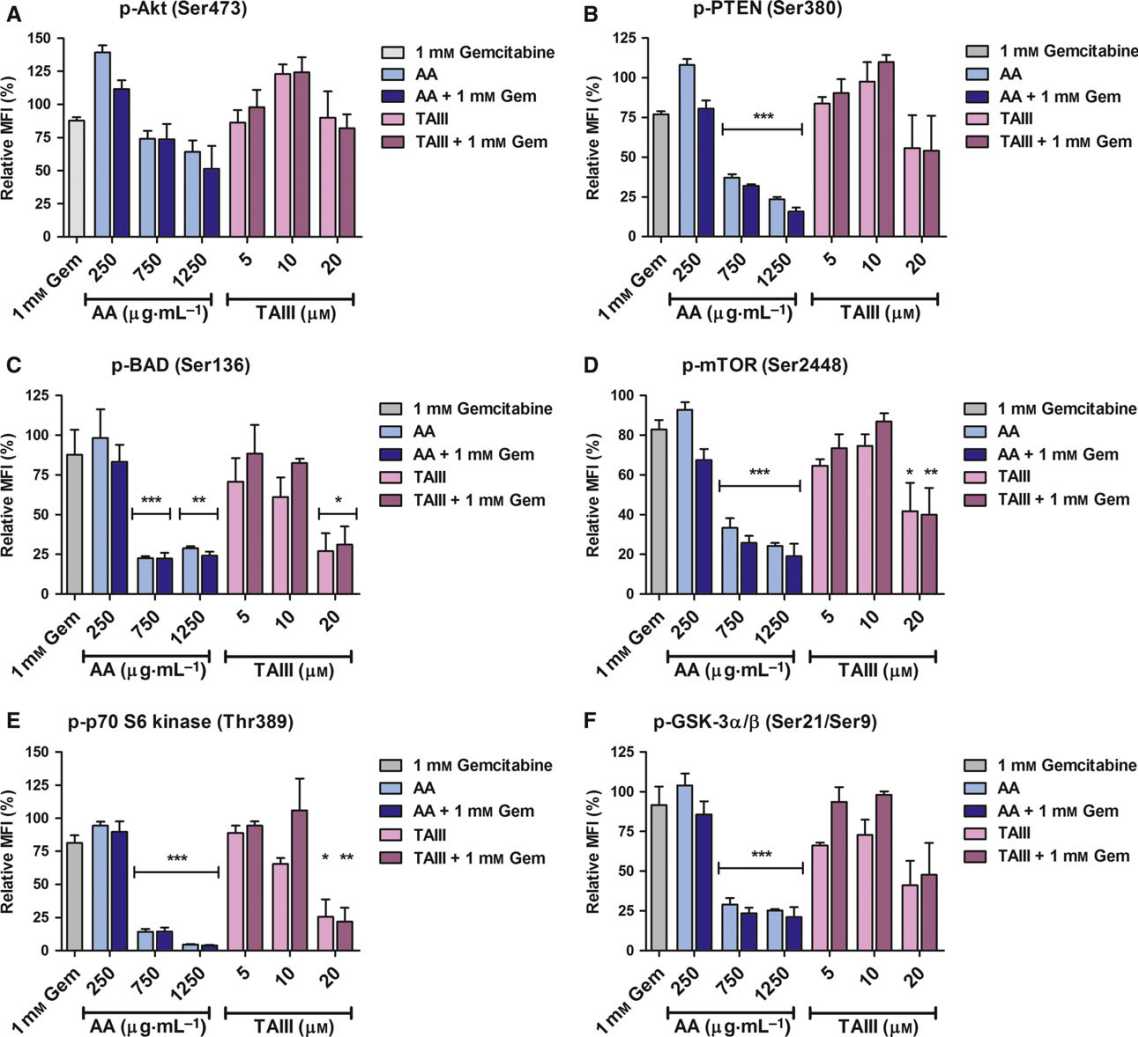
AA and TAIII in the presence and absence of gemcitabine modulate the activity of Akt signaling pathway members. PANC‐1 cells were treated with 1 mm gemcitabine, AA or TAIII (250, 750, or 1250 μg·mL^−1^ and 5, 10, or 20 μm, respectively) alone or with 1 mm gemcitabine, or vehicle for 24 h. Phosphorylation of several Akt pathway members (A–F) was measured by the magnetic‐based cell signaling multiplex assay. Results from two independent experiments are expressed as the relative percent MFI compared to the vehicle‐treated control for each analyte. Significant differences from the gemcitabine‐only treatment are indicated (*P < 0.05, **P < 0.01, ***P < 0.001).

To determine the effect of AA and TAIII on the activation of PI3K/Akt pathway proteins, PANC‐1 cells were treated for 24 h as described above and evaluated for phosphorylated Akt pathway proteins using the Bio‐Plex pro cell signaling Akt panel. Activation via phosphorylation of the pro‐survival proteins mTOR and p70 S6 kinase was significantly decreased in PANC‐1 cells treated with AA alone and with gemcitabine compared to those in which only gemcitabine was administered (*P* < 0.001; Fig. 6D,E). Inhibitory phosphorylation of the pro‐apoptotic proteins PTEN and BAD was also significantly decreased in PANC‐1 cells treated with AA alone and in combination with gemcitabine compared to cells treated with gemcitabine alone (*P* < 0.01 or *P* < 0.001; Fig. 6B,C). While the differences in the expression levels of phosphorylated Akt, PTEN, mTOR, BAD, and GSK‐3 between cells treated with AA and those treated with AA in the presence of gemcitabine did not reach statistical significance, there is a clear stepwise and dose‐dependent decrease. This may indicate that even subtle differences in the activity of these efficient proteins result in a biological effect before reaching levels of statistical consequence. In the instance of phosphorylated p70 S6 kinase, expression does not appear to differ between cells treated with AA alone and with AA in the presence of gemcitabine. It is possible that AA alters the expression of some cellular targets in such a drastic manner that it eclipses any effect gemcitabine may have had. Phosphorylation of BAD, mTOR, and p70 S6 kinase was significantly decreased in cells treated with 20 μm TAIII alone and with gemcitabine compared to gemcitabine alone (*P* < 0.05 or *P* < 0.01; Fig. 6C–E). Similar to the effect seen between treatment with AA and with AA plus gemcitabine, the levels of phosphorylated BAD between cells treated with 20 μm TAIII with and without gemcitabine do not differ statistically, although there may still be a biologically significant variance. Additionally, there may still be other causative proteins targeted by AA and TAIII that we have yet to identify.

### AA and TAIII induce caspase‐dependent apoptosis in PANC‐1 cells

**Figure 7:**
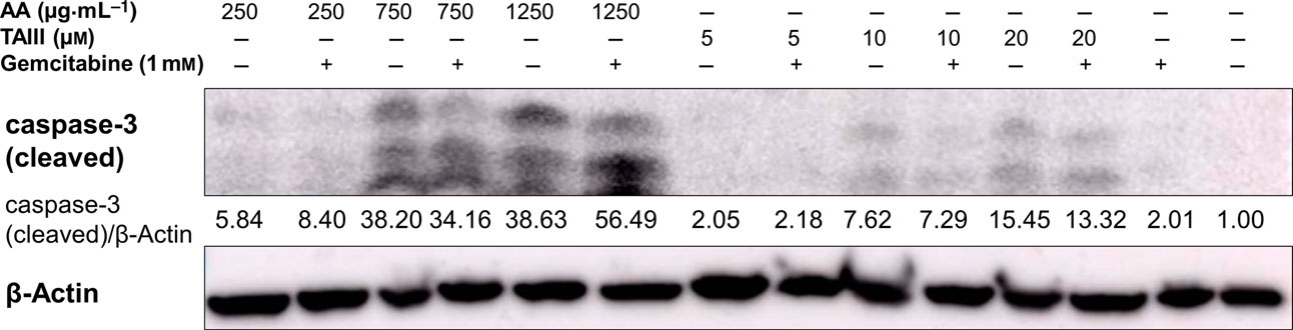
Caspase‐3 activation in PANC‐1 cells treated with AA or TAIII and gemcitabine. The expression of activated caspase‐3 was examined by Western blot analysis of PANC‐1 cells treated with AA or TAIII, alone or in combination with 1 mm gemcitabine, 1 mm gemcitabine alone, or vehicle. Expression of β‐Actin used as a loading control. Data shown are representative of three independent experiments.

Based on the combined results of the viability, cell cycle, and multiplex analysis, we suspected AA and TAIII elicit a caspase‐dependent apoptotic cascade. To investigate this, the lysates of PANC‐1 cells treated with AA or TAIII alone and in the presence of gemcitabine were examined by Western blot analysis for activated caspase‐3. The results showed a clear dose‐dependent increase in active caspase‐3 in PANC‐1 cells treated with AA compared to the vehicle‐treated control cells and those treated with only gemcitabine (Fig. 7). There is a similar trend in the cells treated with increasing concentrations of TAIII, albeit to a lesser degree. Interestingly, the level of active caspase‐3 in the cells treated with TAIII is slightly greater than in the cells treated with both TAIII and gemcitabine which mirrors the cell viability data seen in Fig. 3; again this suggests that the presence of gemcitabine affects the activity of TAIII within the cells. These results indicate that AA and TAIII initiate apoptosis in PANC‐1 cells via a caspase‐dependent mechanism.

## Discussion and conclusion

Pancreatic ductal adenocarcinoma is one of the most highly lethal cancers, and its incidence rate is currently on the rise. Due to a lack of sensitive and accurate detection methods, a propensity for metastasis, and nearly universal drug resistance, the 5‐year survival rate among patients whose tumors have disseminated at the time of detection is among the lowest of any cancer 1, 20. In the three decades since the landmark study that led to gemcitabine as the primary chemotherapeutic agent for PDAC, progress toward a more effective and less toxic treatment has been scant. Recent treatment regimens such as gemcitabine + nab‐Paclitaxel and 5‐fluorouracil, leucovorin, irinotecan, oxaliplatin (FOLFIRINOX) have offered only modest improvements to overall survival or debilitating side effects, and therefore, the dire need for effective treatment with low toxicity still remains 21, 22.

In recent years, interest in natural products such as herbal extracts as anticancer agents has gained significant traction as they offer a multitude of active compounds and the potential for significant reduction in the toxicity associated with traditional chemotherapy. The extract of AA Bunge and its bioactive compound TAIII have demonstrated anticancer effects in a number of cancer types; however, neither has been investigated for efficacy against PDAC cell growth and gemcitabine resistance which we have done in our present study. Our results demonstrate that AA and TAIII markedly inhibit the growth of PANC‐1 and BxPC‐3 cells and induce caspase‐dependent apoptosis by modulating the activity of PI3K/Akt pathway proteins involved in cell cycle and proliferation. As seen in Fig. 2, treatment of PANC‐1 and BxPC‐3 cells with AA exhibits a hormetic‐like, biphasic dose response wherein there is a low‐dose stimulatory effect and subsequent inhibition with increasing doses. This dose‐response phenomenon frequently occurs across biological models as low concentrations of a particular substance elicit a protective stress response within the cells, while higher doses overwhelm normal repair mechanisms and are able to affect the intended receptor or signaling pathway 23, 24. A similar biphasic dose response can be seen in the results of our multiplex analysis examining the expression of phosphorylated Akt and PTEN (Fig. 6A,B). Notably, both AA and TAIII exhibit a greater degree of inhibition in the PANC‐1 cells which harbor an activating G12D Kirsten rat sarcoma viral oncogene homologue (KRAS) mutation than in the BxPC‐3 cells which express wild‐type KRAS 25. The results also reveal that PANC‐1 cells subjected to TAIII with gemcitabine exhibit less inhibition and modulation of PI3K/Akt pathway proteins than those treated with TAIII alone. The opposite trend is true for AA, where treatment with AA in the presence of gemcitabine is more effective than with AA alone. This could indicate that one or more of the processes by which these cells resist gemcitabine also, at least partially, affects TAIII. Several mechanisms of gemcitabine resistance have been demonstrated in PDAC cells and tumors, including highly efficient efflux of the drug due to increased expression of multidrug‐resistant and ATP‐binding cassette transporters which could potentially also affect levels of TAIII within the cells 26, 27.

Regulation of the cell cycle involves a delicate balance of several concerted processes whose disruption is a hallmark of tumor proliferation and drug resistance. Thus, chemotherapeutic agents that can force cancer cells into an arrested cell cycle and eventually induce apoptosis are of great interest. Our results show that AA causes the cells to steadily decrease S and G_2_ phase populations, while the majority of cells enter a sub‐G_1_ phase. Treatment with TAIII leads to a dose‐dependent decrease in S and G_2_ phase populations and increased G_1_ populations, resulting in G_1_ arrest. Multiplex analysis also revealed that treatment with AA led to a significant decrease in phosphorylated GSK‐3α/β and, along with TAIII (20 μm), significantly reduced phosphorylation of p70 S6 kinase in PANC‐1 cells compared to treatment with gemcitabine. p70 S6 kinase is activated by phosphorylation and one of its major functions is regulation of cell growth, and it is required for progression through the G_1_ phase 28. GSK‐3 is a critical downstream kinase in the PI3K/Akt pathway and can be inhibited via Akt‐mediated phosphorylation at Ser21 (GSK‐3α) and Ser9 (GSK‐3β) 9. Under normal conditions, GSK‐3β regulates cyclin D1 turnover and subsequently the transition from G_1_ phase to S phase by phosphorylation; however, the role of GSK‐3α/β in PDAC remains a topic of controversy as it has demonstrated both tumor suppressor and oncogenic effects 29. The activities of GSK‐3 are also highly dependent upon whether it is located in the cytoplasm or nucleus 30. Taken together, these data indicate that AA and TAIII disrupt PDAC cell cycle progression, are concordant with the viability assay data shown in Figs 2 and 3, and suggest the possibility of distinct mechanisms of action between AA and TAIII.

In addition to aberrant cell cycle and growth regulation, PDAC is highly adept at circumventing apoptosis induction by both intracellular mechanisms and chemotherapeutic agents. It has been repeatedly demonstrated that overactivation of the PI3K/Akt pathway contributes to proliferation and drug resistance in PDAC. Thus, this pathway is an attractive target in the search for vulnerabilities within PDAC cells to attenuate resistance and induce apoptosis. Our results indicate that while the combined effect is not synergistic, both AA and TAIII singularly and with gemcitabine are able to inhibit the phosphorylation of PI3K/Akt pathway proteins and promote apoptosis in PDAC cells to a much greater extent than gemcitabine alone.

In summary, the results of our study demonstrate that AA Bunge and its constituent TAIII modulate the activity of PI3K/Akt pathway proteins and are potent inhibitors of PDAC cell proliferation. These findings demonstrate the importance of further investigation into the potential antitumorigenic and gemcitabine‐sensitizing properties of AA and TAIII in treating advanced pancreatic cancer.

## Author contributions

CM, BB, and CZ conceived and designed experiments. CM, AS, and TS performed experiments and data analysis. BB supervised research project. CM and BB wrote the manuscript. All authors read and approved the final manuscript.
